# Perceived Neighbourhood Problems over Time and Associations with Adiposity

**DOI:** 10.3390/ijerph15091854

**Published:** 2018-08-28

**Authors:** Anne Ellaway, Ruth Dundas, Jonathan R. Olsen, Paul G. Shiels

**Affiliations:** 1 MRC/CSO Social and Public Health Sciences Unit, University of Glasgow, 200 Renfield Street, Glasgow G2 3QB, UK; ruth.dundas@glasgow.ac.uk (R.D.); Jonathan.Olsen@glasgow.ac.uk (J.R.O.); 2 Institute of Cancer Sciences, University of Glasgow, Glasgow G61 1QH, UK; paul.shiels@glasgow.ac.uk

**Keywords:** neighbourhood, neighbourhood perceptions, adiposity, obesity, abdominal obesity, longitudinal study, urban environment, life course models

## Abstract

There is growing interest in understanding which aspects of the local environment influence obesity. Using data from the longitudinal West of Scotland Twenty-07 study (*n* = 2040) we examined associations between residents’ self-reported neighbourhood problems, measured over a 13-year period, and nurse-measured body weight and size (body mass index, waist circumference, waist–hip ratio) and percentage body fat. We also explored whether particular measures such as abdominal obesity, postulated as a marker for stress, were more strongly related to neighbourhood conditions. Using life course models adjusted for sex, cohort, household social class, and health behaviours, we found that the accumulation of perceived neighbourhood problems was associated with percentage body fat. In cross-sectional analyses, the strongest relationships were found for contemporaneous measures of neighbourhood conditions and adiposity. When analyses were conducted separately by gender, perceived neighbourhood stressors were strongly associated with central obesity measures (waist circumference, waist–hip ratio) among both men and women. Our findings indicate that chronic neighbourhood stressors are associated with obesity. Neighbourhood environments are modifiable, and efforts should be directed towards improving deleterious local environments to reduce the prevalence of obesity.

## 1. Introduction

The rise in obesity is a growing concern worldwide [1] as it is a key risk factor for non-communicable diseases such as cardiovascular disease, type 2 diabetes, and some cancers [2] as well as reduced longevity [3]. In addition to the health and economic burden to the individuals experiencing obesity, the cost to health-care services and loss of productivity is estimated at over US $2 trillion worldwide [4], with obesity accounting for between 2% and 6% of total health-care costs in many countries [5]. Concerns have been raised that, although the number of premature deaths from cardiovascular disease has been falling in high-income countries over the last 40 years, this trend may be impeded by the rise in obesity and associated diseases such as type 2 diabetes [6,7]. In high-income countries such as the United Kingdom and the United States, obesity is more prevalent among more disadvantaged groups such as those living in poor neighbourhoods [6].

Attempts to address key determinants of obesity, such as initiatives to promote physical activity or a healthy diet directed at individuals, have had limited success to date [8,9]. This has led to increasing efforts to understand environmental influences, particularly those related to the built environment [10]. There is growing interest in the role of the residential environment as a determinant of health and unpacking the features is considered a priority [11]. Understanding the environmental factors that are associated with obesity is key to informing and developing interventions aimed at reducing its prevalence [6].

Some studies have shown that living in more deprived neighbourhoods is associated with obesity, even after individual characteristics such as socio-economic status, gender, and age are taken into account [12,13]. The importance of the residential environment in influencing obesity is further elucidated by findings that moving to deprived neighbourhoods is associated with an increase in obesity [14,15]. One potential mechanism through which area of residence might influence obesity is how residents experience their local environment. A small number of studies have explored this; for example, in a cross-sectional study of English adults, Poortinga found that perceived problems such as vandalism were associated with obesity, and this was not mediated by correlates of obesity including diet and physical activity [16]; and Burdette and Hill found that perceived neighbourhood disorder was associated with an increased risk of obesity among Texan adults [17].

With a few exceptions [18] most studies to date are cross-sectional, which limits knowledge of the pathways linking neighbourhood and obesity. The psychosocial stress associated with living in poor neighbourhood conditions may operate directly or indirectly in ways which influence adiposity. Prolonged stress may directly affect homeostatic mechanisms, in that individuals may experience chronic activation of the hypothalamic–pituitary–adrenal (HPA) axis which releases cortisol, resulting in prolonged elevation of cortisol with energy consequently stored as fat [19]. This is in contrast to short-lived stress where the HPA axis is activated (fight or flight) but returns to normal after the stressful event is over [17]. Stress has been associated with body weight in a number of studies [20,21] and it has been suggested that stress which activates the HPA axis leads to abdominal obesity in particular [22]. Indirect mechanisms related to the experience of poor neighbourhood conditions and correlates of adiposity include coping strategies such as increased emotional or ‘comfort’ eating [23,24], and feeling unsafe may deter physical activity such as walking around the local neighbourhood [25].

Moreover, as most studies to date which have examined perceptions of the neighbourhood and adiposity are cross-sectional, there have been calls to examine the effects of cumulative exposures to adverse neighbourhood conditions on health [18,26]. We set out to examine whether perceived neighbourhood stressors were associated with adiposity using three sweeps of data, covering 13 years of the life course, from three cohorts in the longitudinal West of Scotland Twenty-07 Study. The West of Scotland Twenty-07 Study is a community-based, prospective cohort study, which has followed three cohorts of men and women recruited at the (approximate) ages of 15 (1970s cohort), 35 (1950s cohort), and 55 years (1930s cohort) in 1987 (wave 1) and followed up over 20 years [27].We therefore assessed two models to examine the impact of perceived neighbourhood stressors on adiposity. These comprised: (1) an accumulation model which uses a cumulative measure of neighbourhood perceptions over time; and (2) a critical periods model which separately analyses the relationship between measures of perceived neighbourhood stressors at each wave and subsequent adiposity. As males and females may differ in their experience of their residential environment and their health and health behaviours and there have been calls to better understand this [18], we also examine if relationships between neighbourhood perceptions and adiposity are patterned by gender. Our measures of adiposity are body mass index (BMI), waist–hip ratio, waist circumference (postulated as a more important risk factor for cardiovascular disease [28]), and percentage body fat. Here we assess if particular measures of adiposity such as central obesity (waist, waist–hip ratio) which are implicated in activation of the HPA axis are more strongly related to neighbourhood perceptions compared to overall excess weight reflected by BMI.

## 2. Methods

Data were from the West of Scotland Twenty-07 Study, a community-based prospective cohort study which is situated within a large socially heterogeneous and predominantly urban area surrounding the city of Glasgow [27]. This study area was considered suitable for investigating inequalities in health and the determinants of health because it contains areas with the best and the worst health statuses in Europe [29,30]. At the 2011 census, over 92% of the Scottish population identified themselves as being of ‘white’ ethnicity (80 to 98% within the West of Scotland). Over 40% of the population in some local authority areas in the West of Scotland had a university degree, as compared to other areas where only 16% did. In some areas (e.g., Glasgow city) over 45% of children live in poverty, compared to 5% in other areas [31]. West of Scotland Twenty-07 baseline respondents have been shown to be representative of the general population of the sampled area [32]. The methodology and specific study design of the West of Scotland Twenty-07 have been published and described in detail [27].

The Tayside Committee on Medical Research Ethics approved the study. Informed, written consent was obtained from all respondents at each wave of the study. For the West of Scotland Twenty-07 Study, three cohorts of men and women were recruited in 1987 at the (approximate) ages of 15 (1970s cohort), 35 (1950s cohort), and 55 years (1930s cohort) and followed over 20 years. There are five waves for each cohort (e.g., wave 1—1987/1988; wave 2—1990/1991; wave 3—1995; wave 4—2000/2004; final wave—2007/2008). Here, we examine data from three sweeps of data collection (1995, 2000, 2007) for which we have equivalent exposure variables. Outcome data on adiposity were collected in wave 5 by trained nurses in the homes of the study participants when the mean age of respondents was 36.7 (Standard Deviation [SD] 0.4) for the 1970s cohort, 57.1 (SD 0.8) for the 1950s cohort, and 76.2 (SD (0.6) for the 1930s cohort. At wave 5, 2040 had complete data for sex, cohort, and social class. For physical measures, 21 had data missing for BMI, and 23 had data missing for waist and hip measurements. Thirty-two respondents had data missing on body fat measures. There were no missing values for neighbourhood perceptions. The numbers of respondents who had missing data on the health behaviours measures were: 25 respondents on participation in exercise, 7 respondents for smoking behaviour, and 28 respondents for consumption of sweets and chocolate. Social class at wave 5 was coded according to the Registrar General’s 1980 classification [33] for head of household’s current or previous occupation.

### 2.1. Outcomes

Body weight (clothed) was measured to the nearest 0.5 kg by portable electronic scales calibrated at the local trading standards office. Height was measured to the nearest centimetre using Leicester stadiometers (Seca, Birmingham, UK), with respondents standing without shoes, heels against the wall, and head in the Frankfort plane. BMI was calculated as: weight (kg)/height (m)^2^. Using a metal tape, waist circumference was measured midway between the lower rib margin and the iliac crest; hip circumference was measured at its maximum point over the greater trochanters. Waist–hip ratio was calculated by dividing waist measurement by hip measurement. Body composition was measured using the Bodystat 1500MDD (Bodystat, Isle of Man, Great Britain) [34]. Any woman in the early stages of pregnancy or subjects with pace-makers or any implantable electronic device were excluded from the measure. Body fat percentage was calculated as (body fat (kg)/body weight (kg)) × 100.

### 2.2. Exposures: Perceptions of Neighbourhood Quality

An identical suite of questions on respondents’ perceptions of their local neighbourhood was asked at three waves of the study: wave 3 (1995), wave 4 (2000), and wave 5 (2007). Respondents were asked about six socio-environmental problems (vandalism, litter and rubbish, assaults and muggings, disturbances by children or youngsters, smells and fumes, burglaries) and invited to reply using a three-point scale (‘not a problem’ score 1, ‘minor problem’ score 2, ‘serious problem’ score 3). As we were interested in how respondents who consider that there is a ‘major‘ problem with an issue in their area may vary in their adiposity outcomes compared to those who have more positive perceptions and to provide a more differentiated score on the perceived severity of the problems [35], scores were revised at each wave so that ‘serious problem’ was given a score of one and ‘minor problem’ and ‘not a problem’ were each scored as zero. Neighbourhood perceptions were subsequently coded as 0 (‘none’) or 1 (1–6) for each wave and then this dichotomy was used for the life course approach to the analyses.

### 2.3. Confounders: Health Behaviours

Our models include confounders known to be associated with obesity such as emotional or ‘comfort’ eating (consumption of high fat high sugar foods such as sweets and chocolate), smoking status (current and ever smoking status), and physical activity (number of days per week doing vigorous exercise for 20 or more minutes continuously).

### 2.4. Statistical Analysis

A structured approach to selecting the most appropriate life course model for each outcome was taken [36]. The analyses consisted of two stages; the first stage selected the most appropriate life course model and the second stage examined the effect of the chosen lifetime neighbourhood perception on the four adiposity measures, adjusting for sex, cohort, household social class and health behaviours (exercise, smoking and consumption of ‘comfort’ food such as sweets and chocolate). The analyses steps were repeated for men and women separately. Adiposity measures were tested for skewness and all were normally distributed, with the exception of BMI which was very slightly skewed but not sufficiently to warrant different treatment of this variable.

The structured life course approach compares models nested within a saturated model where all the eight possible combinations of poor neighbourhood perception are related to the outcome of interest. This is equivalent to fitting a linear regression with full factorial design (i.e., three main effects of period, three two-way interactions between time periods, and the three-way interaction of time periods). The accumulation model comprised main effects of the three time periods only, not interactions. The assumption was each wave could have a variable contribution to the effect; the waves were not constrained to having an equal effect on the outcome [37]. There were three critical period models, one for each period. The critical period models assume that the neighbourhood perception only contributes to adiposity for that period, irrespective of the other time periods.

Each model was compared to the saturated model using a partial F-test. A higher *p*-value means the model is not different from the saturated model and thus it should be selected as the ‘best’ model. The F-test can be used to compare models that are nested. The critical period models are not nested within each other and so the Bayesian Information Criterion was used to select the best model.

Once the best model was selected for each adiposity outcome, that model was adjusted for sex, cohort, and household social class, with further adjustment for dietary intake of ‘comfort’ foods (sweets and chocolate), physical activity, and smoking.

Analyses were conducted in StataSE (ver14) (StataCorp, Texas, TX, USA).

## 3. Results

Table 1 shows the basic distributions of adiposity and the four measures of neighbourhood perceptions. Among our male respondents, mean BMI ranged from 16.21 to 63.44 ($\bar{x}=$ 28.0); among female respondents, BMI ranged from 15.65 to 58.64 ($\bar{x}=$ 28.0). Males had significantly higher waist circumferences and waist–hip ratios compared to females, however females had higher body fat percentages than males. Waist circumference ranged from 54.0 to 176.0 ($\bar{x}=$ 0.94) in men, and from 50.85 to 152.10 ($\bar{x}=$ 90.7) in women. Waist–hip ratio ranged from 0.76 to 1.22 ($\bar{x}=0.94$) in men, and from 0.63 to 1.10 ($\bar{x}=$ 0.85) in women. Table 2 shows the mean (SD) adiposity outcomes by neighbourhood perceptions permutation.

Table 3 shows the *p*-values and Bayesian Information Criterion to assess the selection of lifecourse model for the relationship of neighbourhood perceptions over time with BMI, waist–hip ratio, waist circumference, and fat percentage for all respondents (Table 3), for males (Table 4), and for females (Table 5). For all respondents taken together, the accumulation model was the best-fitting model for BMI and fat percentage, critical period wave 4 was best-fitting model for waist circumference, and the best-fitting model for the waist–hip ratio was critical period wave 5. For men, the best–fitting model for BMI, was the critical period wave 3 model. For waist circumference, waist–hip ratio, and body fat percentage, critical period wave 5 was the best fitting model. For women, the accumulation model was the best-fitting for BMI, waist circumference, and body fat percentage, and critical period wave 4 was the best-fitting model for waist circumference.

We then examined the relationships between neighbourhood stressors and adiposity, controlling for health behaviours (exercise, smoking, and consumption of sweets and chocolate). For all respondents taken together (Table 6) there was a significant relationship in the model for accumulation of neighbourhood stressors for percentage body fat; cross-sectional associations were found at wave 5 between neighbourhood stressors and BMI and waist–hip ratio. At wave 4 neighbourhood stressors were statistically significant for waist circumference. When we examined the relationships separately for males and females, among men (Table 6), statistically significant relationships were observed for neighbourhood stressors and waist–hip ratio at wave 5, and among women, in waist circumference at wave 4.

## 4. Discussion

We examined the relationship between perceived neighbourhood conditions and adiposity and found that more negative perceptions were associated with higher adiposity. We found stronger relationships for abdominal obesity measures and percentage body fat, and weaker relationships with BMI, providing some support for a mechanism whereby prolonged stress might activate the HPA axis and in turn lead to increased abdominal obesity. Our findings contribute to furthering our understanding of the determinants of adiposity, particularly abdominal obesity, which, as noted earlier, is a risk factor for cardiovascular disease, type II diabetes, and stroke [38,39]. BMI may be a poorer measure of obesity than our other measures given that it cannot distinguish between fat mass and muscle mass, nor capture the distribution of fat in the body [40].

Our findings are novel and add to the small body of work examining the relationships between perceived neighbourhood conditions and obesity, such the study by Kwarteng et al., who found that perceptions of the neighbourhood physical environment mediated the relationship between neighbourhood poverty and central adiposity [41]; and Pham et al., who found that perceived neighbourhood safety was associated with abdominal obesity among women but not men [42]. In our study, we found central obesity measures to be independently associated with neighbourhood stressors among both men (waist–hip ratio) and women (waist circumference) when examined separately.

In the literature, most studies, including ours, have found that poor neighbourhood conditions are more prevalent in deprived areas, and it has been suggested that the potential health harms that may accrue from living in a deprived area may be ‘buffered’ by strong social ties and supportive relationships [43]. However, although we did not examine this factor in the present study, we have previously found that a lack of social cohesion may be more common in deprived areas and may produce poorer mental health [44]. Living alongside others who are also experiencing similar stressful environments may reduce the possibility of building strong communities. Moreover, living in a disadvantaged neighbourhood with few opportunities to access healthy foods has been shown to be associated with a poorer diet [45]. Another possibility is that people in poor quality neighbourhoods may turn to comfort or emotional eating to alleviate stress [23,24]. However, our models included measures of a poor diet/comfort eating (consumption of high-fat high sugar foods such as crisps, sweets, and chocolate).

The strengths of our study include that it used a large well-characterised sample of a wide age range, objectively measured adiposity, and data on neighbourhood perceptions over time to explore the contribution of prolonged exposure to poor neighbourhood conditions to obesity. Most studies examining this issue are based on self-reported obesity measures (e.g., Burdette and Hill [17]). A potential weakness of our findings that perceived neighbourhood problems are associated with adiposity is that this may be subject to residual confounding in that there are likely to be unmeasured socioeconomic circumstances that affect these associations, particularly those measured across the life course [46]. Finally, although we have found that chronic environmental stressors, such as neighbourhood problems accumulated over time, are related to adiposity, we do not have a measure of physiological stress in our study and hence are unable to examine direct biological pathways between neighbourhood conditions and adiposity.

## 5. Conclusions

In conclusion, we have shown that chronic neighbourhood stressors are associated with obesity, particularly central obesity measures. Neighbourhood environments are modifiable, and efforts should be directed towards improving deleterious local environments to reduce the prevalence of obesity. Moreover, it has been noted that tackling obesity requires a whole systems approach [47]. Our findings that the ways in which people experience their local neighbourhood is associated with obesity highlight that a range of collaborative strategies by different agencies is needed (e.g., from local authorities responsible for street cleaning and maintenance of public spaces). Upgrades may in turn may improve residents’ perceptions of their neighbourhood, encouraging them to move around their local area more and increase social cohesion, thereby potentially reducing stress from poor neighbourhood quality. Public health practitioners’ should attempt to reduce obesity by taking cognisance of local conditions and working jointly with local authorities. Lastly, our findings provide useful evidence for key stakeholders such local community groups and activists aiming to improve their neighbourhoods.

## Figures and Tables

**Table 1:** Basic descriptives. BMI: body mass index.

	n	BMI	Waist–Hip Ratio	Waist	% Body Fat
		$\bar{x}\;(\mathrm{Range})$	$\bar{x}\;(\mathrm{Range})$	$\bar{x}\;(\mathrm{Range})$	$\bar{x}\;(\mathrm{Range})$
Sex			*p* < 0.001	*p* < 0.001	*p* < 0.001
Males	924	28.0 (16.21–63.44)	0.94 (0.7–1.22)	99.3 (54.0–176.0)	29.0 (12.7–47.80)
Females	1106	28.0 (15.6–58.64)	0.85 (0.6–1.10)	90.7 (60.8–152.10)	39.5 (12.7–57.79)
Cohort			*p* < 0.001	*p* < 0.001	*p* < 0.001
1970s	736		27.8	0.87	32.7
1950s	829		28.4	0.90	35.2
1930s	475		27.6	0.91	36.8
Head of Household Social Class				*p* < 0.001	*p* < 0.001
I	255		27.4	93.6	33.0
II	849		27.8	94.0	34.2
III non-manual	424		28.3	95.0	35.7
III manual	278		28.4	96.2	34.4
IV	181		28.2	95.4	36.7
V	53		27.9	96.1	35.8
Total	2040				

**Table 2:** Mean (Standard Deviation) adiposity outcomes by neighbourhood perceptions permutation.

		Perception ^a^		n	BMI	Waist–Hip Ratio	Waist	Fat %
	Wave 3	Wave 4	Wave 5					
Null	0	0	0	1212 (56.2)	27.7 (5.2)	0.89 (0.08)	94.2 (13.26)	34.2 (7.77)
W3	1	0	0	225 (10.4)	28.0 (5.2)	0.88 (0.08)	93.8 (13.86)	34.3 (7.81)
W4	0	1	0	141 (6.5)	28.3 (5.4)	0.88 (0.07)	94.6 (12.03)	35.6 (8.59)
W5	0	0	1	194 (9.0)	28.3 (5.7)	0.89 (0.08)	94.8 (14.06)	34.7 (8.31)
W3 + W4	1	1	0	109 (5.1)	28.4 (6.2)	0.90 (0.08)	96.7 (16.11)	35.0 (8.10)
W3 + W5	1	0	1	81 (3.8)	28.6 (5.7)	0.90 (0.08)	96.6 (13.85)	37.7 (7.31)
W4 + W5	0	1	1	77 (3.6)	29.1 (5.9)	0.90 (0.09)	97.6 (16.18)	36.8 (8.19)
W3 + W4 + W5	1	1	1	117 (5.4)	28.3 (4.9)	0.89 (0.07)	95.2 (13.13)	35.8 (8.45)

^a^ 1 refers to reporting serious neighbourhood problems, 0 refers to minor/no neighbourhood problems.

**Table 3:** *p*-Values and Bayesian Information Criterion (BIC) to assess the selection of the life course model for the relationship of neighbourhood perceptions over time with body mass index (BMI), waist–hip ratio, waist circumference, and fat percentage (FOR THE OVERALL SAMPLE).

	BMI			Waist–Hip Ratio			Waist			Fat %		
	*p*-Value from F-Test	BIC	∆BIC (Saturated—Life Course)	*p*-Value from F-Test	BIC	∆BIC (Saturated—Life Course)	*p*-Value from F-Test	BIC	∆BIC (Saturated—Life Course)	*p*-Value from F-Test	BIC	∆BIC (Saturated—Life Course)
No Effect												
Saturated Model		13,345.49			−4627.77			17,384.18			**14,246.88**	
Accumulation	**0.684**	13,341.09	4.4	0.138	−4627.26	−0.51	0.377	17,380.56	3.62	**0.088**	14,248.11	−1.23
Critical Period (wave 3)	0.221	13,343.16	2.33	0.122	−4627.96	0.19	0.150	17,381.74	2.44	0.002	14,256.28	−9.4
Critical Period (wave 4)	0.493	13,340.45	5.04	0.152	−4628.88	1.11	**0.403**	**17,378.11**	**6.07**	0.019	14,250.95	−4.07
Critical Period (wave 5)	0.560	**13,339.69**	**5.8**	**0.286**	**−4630.81**	**3.04**	0.336	17,378.79	5.39	0.036	14,249.34	−2.46

Numbers in bold indicate the best fitting model.

**Table 4:** *p*-Values and Bayesian Information Criterion to assess the selection of the life course model for the relationship of neighbourhood perceptions over time with BMI, waist–hip ratio, waist circumference, and fat percentage (MEN ONLY).

	BMI			Waist–Hip Ratio			Waist			Fat %		
	*p*-Value from F-Test	BIC	∆BIC (Saturated—Life Course)	*p*-Value from F-Test	BIC	∆BIC (Saturated—Life Course)	*p*-Value from F-Test	BIC	∆BIC (Saturated—Life Course)	*p*-Value from F-Test	BIC	∆BIC (Saturated—Life Course)
No Effect												
Saturated Model		5699.01			−2728.59			7520.29			5768.83	
Accumulation	0.198	5694.88	4.13	0.113	−2728.64	0.05	0.281	7518.37	1.92	0.179	5767.37	1.46
Critical Period (wave 3)	**0.3275**	**5681.75**	17.26	0.020	−2725.03	−3.56	0.297	7515.81	4.48	**0.276**	**5764.19**	**4.64**
Critical Period (wave 4)	0.215	5692.97	6.04	0.013	−2723.84	−4.75	0.183	7517.42	2.87	0.183	5765.69	3.14
Critical Period (wave 5)	0.279	5691.74	7.27	**0.159**	**−2730.79**	2.2	**0.392**	**7514.76**	5.53	**0.285**	**5764.21**	**4.62**

Numbers in bold indicate the best fitting model.

**Table 5:** *p*-Values and Bayesian Information Criterion to assess the selection of the life course model for the relationship of neighbourhood perceptions over time with BMI, waist–hip ratio, waist circumference, and fat percentage (WOMEN ONLY).

	BMI			Waist–Hip Ratio			Waist			Fat %		
	*p*-Value from F-Test	BIC	∆BIC (Saturated—Life Course)	*p*-Value from F-Test	BIC	∆BIC (Saturated—Life Course)	*p*-Value from F-Test	BIC	∆BIC (Saturated—Life Course)	*p*-Value from F-Test	BIC	∆BIC (Saturated—Life Course)
No Effect												
Saturated Model		7568.35			−2800.43			9577.73			7297.91	
Accumulation	**0.726**	7561.96	6.39	0.039	−2797.99	−2.44	**0.448**	**9573.77**	3.96	**0.558**	7293.58	4.33
Critical Period (wave 3)	0.259	7563.62	4.73	0.002	−2785.54	−14.89	0.012	9582.29	−4.56	0.141	7295.74	2.17
Critical Period (wave 4)	0.665	**7559.78**	**8.57**	0.030	−2798.13	−2.3	**0.407**	**9572.00**	**5.73**	**0.519**	**7291.51**	**6.4**
Critical Period (wave 5)	0.600	7560.48	7.87	0.003	−2792.38	−8.05	0.065	9577.59	0.14	0.302	7293.21	4.7

Numbers in bold indicate the best fitting model.

**Table 6:** Adjusted mean differences (95% CI) in main study outcomes for the strongest-fitting life course model, adjusted for health behaviours (exercise, smoking, and consumption of sweets and chocolate).

BMI	Accumulation	2128	0.56 (−0.18 to 1.14)	0.057	Critical Period (Wave 3)	964	0.57 (−0.09 to 1.23)	0.089	Accumulation	1164	0.70 (−0.15 to 1.55)	0.105
	Critical Period (wave 5)	2128	0.71 (0.15 to 1.26)	0.012					Critical Period (wave 4)	1164	0.74 (−0.07 to 1.56)	0.074
Waist–Hip Ratio	Critical Period (wave 5)	2128	0.01 (0.00 to 0.02)	0.005	Critical Period (wave 5)	964	0.01 (0.001 to 0.020)	0.025	Critical Period (wave 5)	1164	0.01 (−0.00 to 0.02)	0.07
Waist	Critical Period (wave 4)	2128	1.79 (0.45 to 3.14)	0.009	Critical Period (wave 5)	964	1.53 (−0.29 to 3.35)	0.099	Accumulation	1164	1.68 (−0.31 to 3.67)	0.098
									Critical Period (wave 4)	1164	2.66 (0.75 to 4.57)	0.006
Fat %	Accumulation	2013	0.65 (0.00 to 1.30)	0.049	Critical Period (wave 3)	921	0.65 (−0.12 to 1.41)	0.097	Critical Period (wave 4)	1092	0.79 (−0.12 to 1.70)	0.089
					Critical Period (wave 5)	921	0.56 (−0.27 to 1.39)	0.183				
